# A Case of Granuloma Annulare Associated with Secukinumab Use

**DOI:** 10.1155/2017/5918708

**Published:** 2017-05-22

**Authors:** Lauren Bonomo, Sara Ghoneim, Jacob Levitt

**Affiliations:** ^1^Department of Dermatology, Icahn School of Medicine at Mount Sinai, 5 East 98th Street, 5th Floor, New York, NY 10029, USA; ^2^Saba University School of Medicine, The Bottom, Saba, Netherlands

## Abstract

Granuloma annulare (GA) is a benign inflammatory dermatosis characterized clinically by dermal papules and annular plaques. The pathogenesis of GA is not well understood, although it is thought to result from a delayed-type hypersensitivity reaction in which inflammatory cells elicit connective tissue degradation. This condition has been seen following the use of several drugs, including tumor necrosis factor-alpha (TNF-*α*) inhibitors, which paradoxically have also been reported to treat GA. We report the case of a patient who developed GA in association with secukinumab, an interleukin-17A antagonist, and discuss its implications for our understanding of the pathogenesis of GA.

## 1. Introduction

Granuloma annulare (GA) is a benign inflammatory dermatosis characterized clinically by dermal papules and annular plaques. The characteristic histopathological finding is a lymphohistiocytic granuloma associated with varying degrees of connective tissue degeneration and mucin deposition. The pathogenesis of GA is not well understood, although it is thought to result from a delayed-type hypersensitivity reaction in which inflammatory cells elicit connective tissue degradation [1].

A number of events predisposing to GA have been reported, including mild trauma, various infections, diabetes mellitus, thyroid disease, and malignancy. Additionally, GA has occurred in patients treated with certain drugs, particularly tumor necrosis factor-alpha (TNF-*α*) inhibitors [2]. We report the case of a patient who developed GA in association with the IL-17A antagonist secukinumab and discuss the implications of this case for our understanding of the pathogenesis of GA.

## 2. Case Report

A 60-year-old Hispanic woman with a medical history of fibromyalgia, hypothyroidism, and Ménière's disease has been treated in our clinic for psoriasis and psoriatic arthritis since 2006. Over the past ten years, the patient has failed topical therapies, etanercept, infliximab, adalimumab, golimumab, and, most recently, apremilast. Upon failure of apremilast in February 2016, the decision was made to attempt therapy with secukinumab. Her other medications at the time included methotrexate, levothyroxine, omeprazole, and duloxetine. Of note, the patient has a self-reported history of hives after treatment with hydrocodone and ibuprofen.

**Figure 1:**
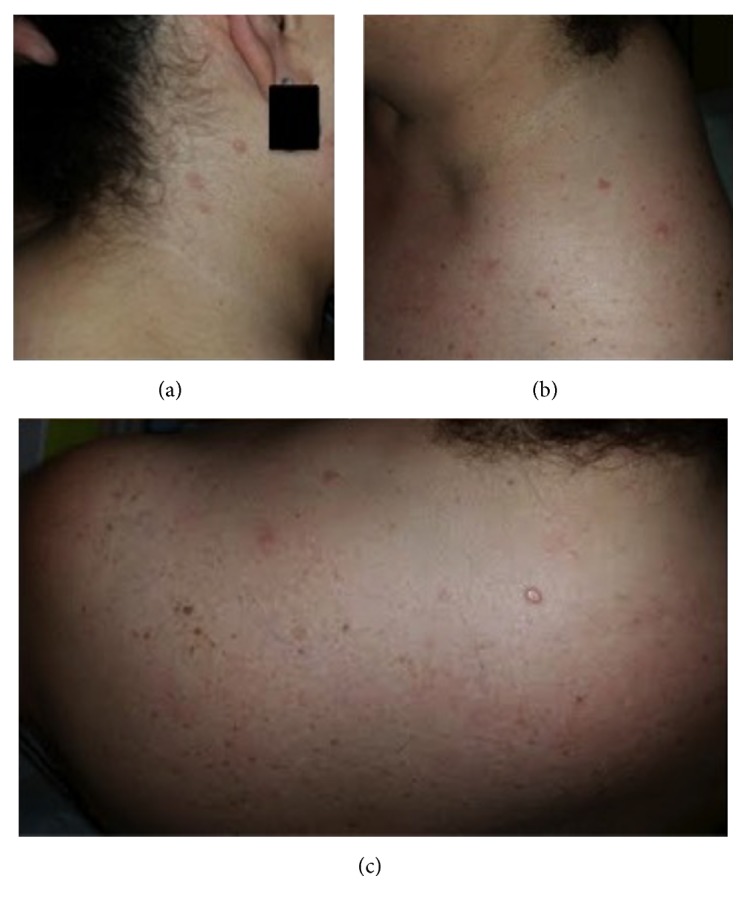
The patient on initial presentation. Tan papules were noted on (a) the right neck, (b) the left neck, and (c) the superior back and shoulders.

The patient received her first dose of secukinumab in April 2016, and improvement was noted in both her psoriatic and arthritic symptoms. However, the patient presented in June with concerns that her psoriasis was beginning to return due to “new” spots on the shoulder, face, and neck. She first noticed the spots approximately two weeks after beginning secukinumab. On examination, the patient was found to have scattered tan papules of the neck, back, and shoulders bilaterally (Figure 1).

**Figure 2:**
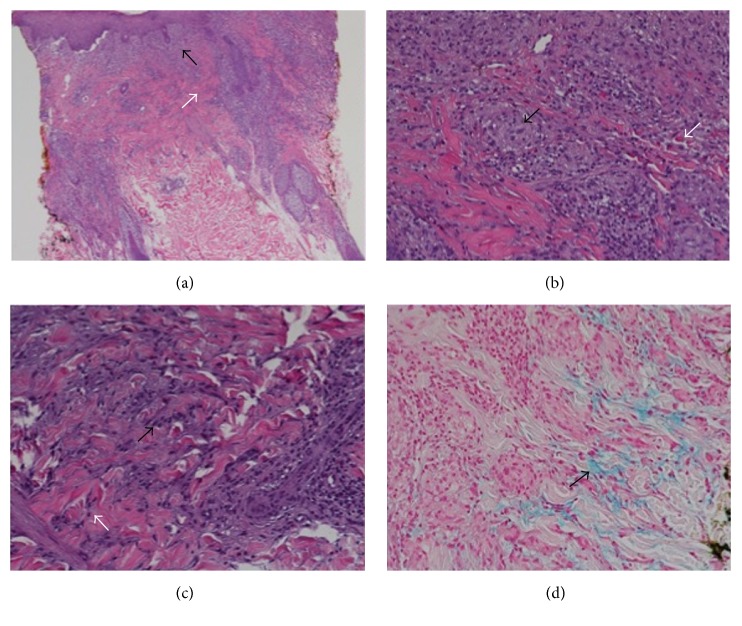
Histopathological slides. At 2x magnification (a), there are superficial periadnexal and interstitial granulomatous infiltrates (black arrow). The epidermis is slightly attenuated with subjacent papillary dermal fibrosis (white arrow). At 20x magnification, the superficial dermis (b) contains well-formed granulomas (black arrow) and individual histiocytes that are scaffolded in between collagen bundles; scattered eosinophils are present (white arrow). In the mid dermis (c), there are numerous interstitial histiocytes in small clusters (black arrow) and solitary units (white arrow). Colloidal iron stain (d) reveals interstitial deposition of dermal mucin (black arrow).

A three-millimeter punch biopsy was taken from a lesion on her right back for histopathological examination. The specimen showed a superficial dermal scar and underlying dermis containing prominent histiocytes with polygonal and cuboidal cytoplasm, in addition to collagen bundles of the superficial and mid dermis (Figure 2). Colloidal iron stain revealed increased dermal mucin. This pattern of inflammation with interstitial histiocytes, focal collagen degeneration, and mucin deposition is consistent with a diagnosis of GA.

The patient was treated with topical clobetasol propionate (0.05%) with mild improvement in lesion size after two weeks. She continued to use secukinumab with no additional adverse events. However, when she returned to our clinic in August, there was no further clinical improvement noted and secukinumab was discontinued. Over the past eight months, the patient did not receive any monoclonal antibody therapy and has reported marked improvement in the GA lesions present on her face and neck.

## 3. Discussion

GA has been associated with a variety of predisposing factors. These include chronic conditions (e.g., diabetes mellitus and thyroid disease), infectious diseases (e.g., human immunodeficiency virus (HIV), Epstein-Barr virus (EBV), varicella zoster virus (VZV), and tuberculosis), minor traumas (e.g., bee stings and sun burn), and various malignancies. Our patient was diagnosed with chronic lymphocytic thyroiditis in 2014; however, she has been euthyroid on levothyroxine since that time. Previous case reports of GA associated with hypothyroidism have shown resolution of the lesions upon treatment with synthetic thyroid hormone [3, 4]. Our patient's chronic medical conditions also included fibromyalgia and psoriatic arthritis. Fibromyalgia is a disorder of pain regulation with no obvious abnormalities on physical examination, while GA is an inflammatory dermatosis. A thorough review of the relevant literature shows no evidence to support the association between fibromyalgia and GA. Likewise, the patient was prescribed duloxetine in 2014 to manage her fibromyalgia symptoms. We believe it is unlikely for duloxetine to be an inciting factor for GA given the well-established safety and adverse profile of the drug. Cutaneous adverse events reported with duloxetine use are rare and include urticaria, contact dermatitis, and Stevens-Johnson syndrome [5]. Psoriatic arthritis (PsA) is an inflammatory arthritis seen in up to 30% of patients diagnosed with psoriasis [6]. While GA and PsA are both inflammatory processes characterized by increased expression of TNF-alpha and matrix metalloproteinases by activated macrophages [7–9], there are no reports to date supporting the role of PsA in the pathogenesis of GA. Further studies are warranted to determine whether the aberrant immunologic signaling observed in psoriasis or PsA plays a direct role in the pathogenesis of GA. Moreover, the patient's psoriasis and PsA symptoms were managed in our clinic since 2006 but the lesions were only observed by the patient two weeks after the administration of secukinumab. Additionally, prior to initiating secukinumab, she was prescribed methotrexate for eight years. A previous case report demonstrated the successful treatment of disseminated GA with methotrexate in part due to the medication's anti-inflammatory properties [10]. Therefore, the GA lesions observed are less likely associated with the above-mentioned chronic conditions and medications and more likely associated with the use of secukinumab.

GA has also been seen following the use of multiple drugs, such as gold therapy, allopurinol, diclofenac, quinidine, intranasal calcitonin, and amlodipine. In 2008, Voulgari et al. demonstrated an association between GA and the use of novel biologic agents. This occurred in nine out of 199 patients receiving infliximab, adalimumab, or etanercept for rheumatoid arthritis. Whereas infliximab, adalimumab, and etanercept target TNF-alpha, secukinumab is a high-affinity, human immunoglobulin G1 monoclonal antibody that selectively binds to and neutralizes interleukin-17A (IL-17A). To our knowledge, this is the first published report of a patient developing GA after treatment with secukinumab. The IL-17A inhibitor was initially approved in 2015 for the treatment of plaque psoriasis but has since been approved for use in psoriatic arthritis and ankylosing spondylitis [11, 12]. The most common adverse events reported with secukinumab are nasopharyngitis, diarrhea, and upper respiratory infection. The only previously reported dermatologic side effects of secukinumab are urticaria and infection.

The pathogenetic mechanisms of GA are poorly understood. One proposed mechanism is that expression of TNF-alpha and certain matrix metalloproteinases by activated macrophages results in matrix degradation [7]. This seems to be supported by evidence that recalcitrant disseminated GA can be successfully treated with the TNF-alpha inhibitor infliximab [13]. However, the work of Voulgari et al. demonstrating a significant association between TNF-alpha antagonist use and development of GA implies that the mechanism may be more complicated or that multiple pathways may be involved. Despite decreasing activity of TNF itself, antagonists may upregulate T-helper 1 lymphocytes, which in turn activate macrophages to produce inflammation and tissue degradation. Our finding that an IL-17A antagonist can also provoke GA formation may provide additional evidence for a T-helper 1 cell-mediated process. Further molecular and immunologic studies are needed to determine whether this is the mechanism by which IL-17A blockade produces this effect.
